# Sister Mary Joseph Nodule and Concomitant Pancreatitis as Initial Presentation of Pancreatic Adenocarcinoma – Case Report and Review of the Literature

**DOI:** 10.7759/cureus.20069

**Published:** 2021-12-01

**Authors:** Murali Dharan, Courtenay Ryan-Fisher

**Affiliations:** 1 Gastroenterology and Hepatology, University of Connecticut Health Center, Farmington, USA; 2 Internal Medicine, University of Connecticut Health Center, Farmington, USA

**Keywords:** pancreatitis, pancreas cancer metastases, metastases, sister mary-joseph nodule, unresectable pancreatic cancer

## Abstract

Umbilical metastasis [Sister Mary Joseph Nodule (SMJN)] is a rare presentation of visceral abdominal/pelvic malignancy. It is less commonly seen in metastatic pancreatic adenocarcinoma and there are only over a hundred cases to date in the literature on this topic. This article highlights a case of metastatic pancreatic adenocarcinoma presenting as SMJN and concomitant pancreatitis (which to the best of our knowledge is the first such report to date) with discussions regarding the etiopathogenesis of this phenomenon and presents a brief literature review on pancreatic adenocarcinoma presenting as SMJN.

## Introduction

We present a case of a patient presenting with a refractory umbilical rash and generalized abdominal pain. Further evaluation revealed elevated lipase, an umbilical nodule, and a large pancreatic cyst on cross-sectional imaging. Histopathologic results were consistent with metastatic pancreatic adenocarcinoma - presenting at Sister Mary Joseph's nodule (SMJN) and concomitant pancreatitis (this is the first such report to date). We conducted a literature review on pancreatic cancer presenting as SMJN including epidemiology, clinical presentation, pathophysiology, diagnosis, and management.

## Case presentation

**Figure 1:**
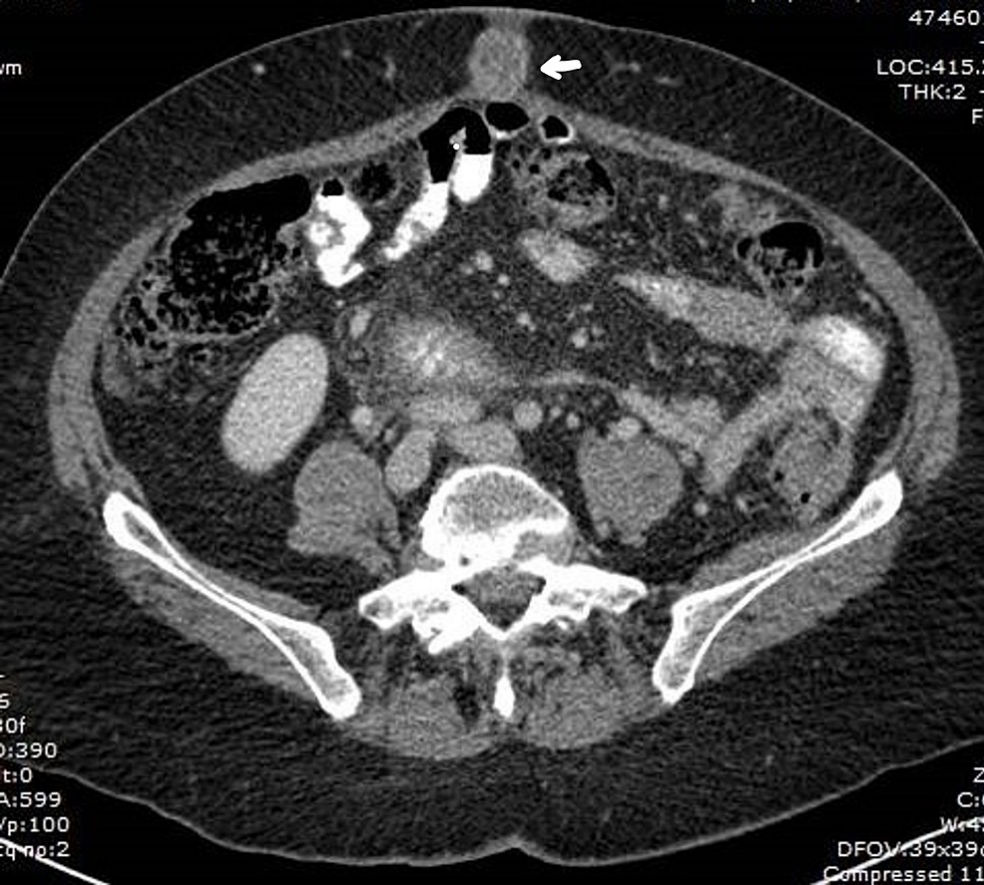
CT scan showing umbilical lesion

**Figure 2:**
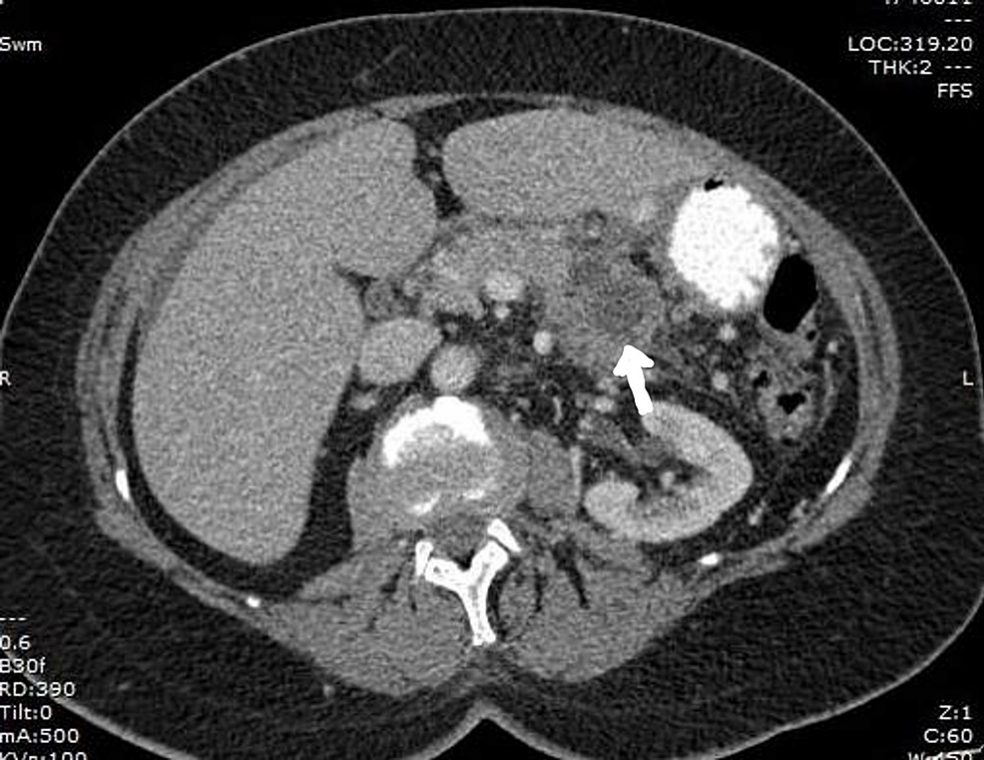
CT scan showing pancreatic tumor in the tail

**Figure 3:**
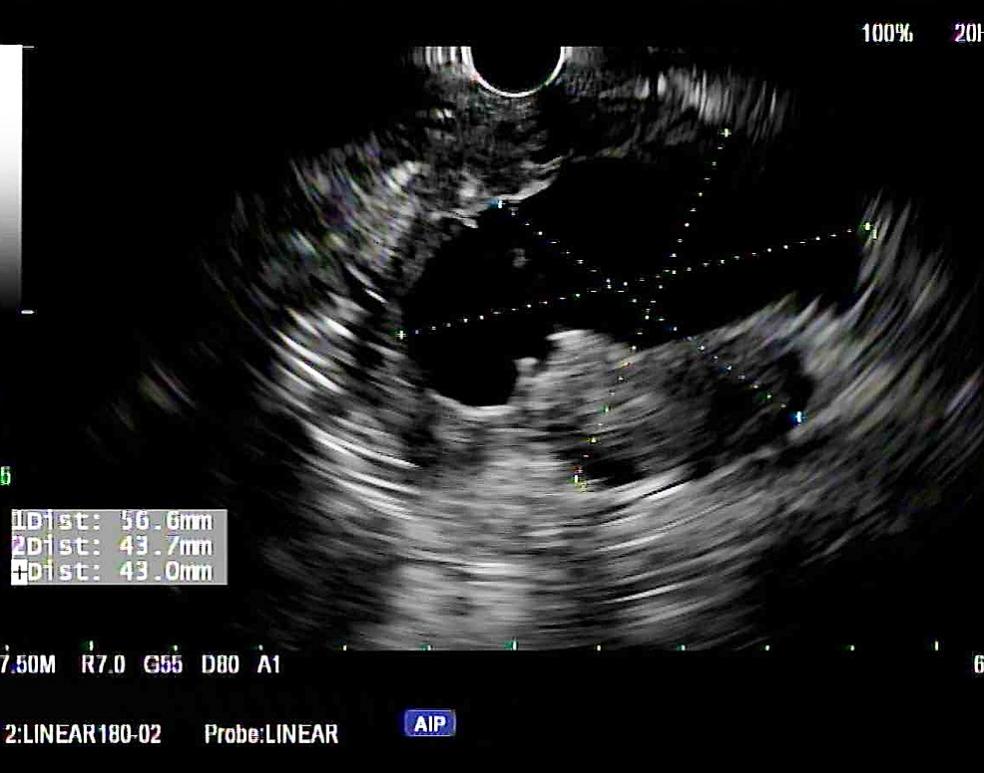
Endosonography (EUS) image of pancreatic lesion

**Figure 4:**
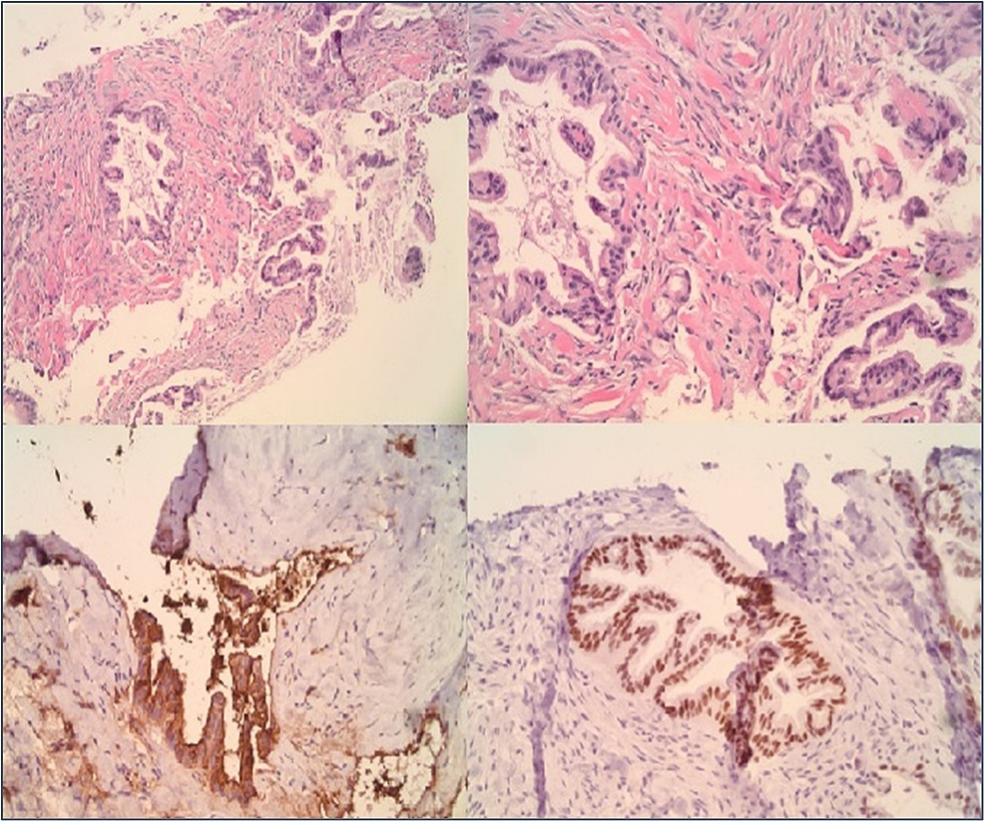
Core biopsy of the umbilical nodule Immunohistochemistry (IHC) positive for pankeratin, CK 7, CA 19-9, CEA, CDX2, CK 19 and negative for CK 20, TTF-1, Napsin A, PAX8.

A 71-year-old female without any significant past medical history presented with two weeks of nausea, generalized abdominal pain, and a peri-umbilical rash. The abdominal pain was localized to the epigastrium and radiated to both the flanks. It was constant and was relieved by narcotics. Associated nausea without vomiting was reported. The pain was not aggravated by food ingestion. The rash was non-tender and non-pruritic. On inspection, an erythematous, indurated, non-tender, palpable peri-umbilical rash with purulent malodorous discharge was appreciated. The patient reported that she had completed a course of antibiotics (for presumed cellulitis) without improvement. Lab work was pertinent for lipase of 2200 u/L. CT abdomen revealed a 5-cm cystic mass of the pancreatic body and tail and a 9-mm fluid collection adjacent to the umbilicus (Figures 1, 2). Endosonography (EUS) revealed a hypoechoic, septated, mixed solid and cystic lesion in the pancreatic body (Figure 3). Fine needle aspiration (FNA) showed highly atypical cells seen in a background of necrosis and cyst fluid. Carcinoembryonic antigen (CEA) and serum carbohydrate antigen 19-9 (CA 19-9) levels were elevated at 6950 u/L and 1113 u/L, respectively. Core needle biopsy of the umbilical mass revealed malignant cells and immunohistochemistry (IHC) was consistent with pancreatic adenocarcinoma (Figure 4). Immunostaining was pankeratin positive, cytokeratin 7 (CK7) positive and cytokeratin 20 (CK 20) negative and strongly CA 19-9 positive suggestive of pancreatic origin. In addition, immunostaining for thyroid transcription factor 1 (TTF-1), Napsin A, and paired box gene 8 (PAX8) was negative. Further workup was consistent with stage IV pancreatic adenocarcinoma. The patient was started on palliative chemotherapy. However, she passed away a few weeks later due to the progression of the disease.

## Discussion

Sister Mary Joseph Nodule (SMJN) refers to cutaneous metastases to the umbilicus from an abdominopelvic malignancy. The most common associated primary sites are the stomach, ovaries, colon, and rectum. Pancreatic cancer presenting as SMJN is rare accounts for 7 to 9% of all cases of SMJN [1]. Less than 100 cases were in the English and Japanese literature as of 1998 [1, 2] and only a few more have been reported since. Other skin manifestations of pancreatic cancer include Trousseau syndrome (a migratory superficial thrombophlebitis of the lower extremities); Leser-Trélat syndrome (multiple seborrheic keratoses on the trunk and extremities); and paraneoplastic hypertrichosis lanuginosa acquisita (lanugo hairs on the head, trunk, and extremities).

Pancreatic cancer commonly spreads to the regional lymph nodes, liver, lungs, celiac plexus, superior mesenteric vessels, portal vein, peri-pancreatic fat, and the skin. While cutaneous metastases are usually a late manifestation of abdominal and pelvic tumors, umbilical metastases are often an early presenting sign of underlying malignancy.

Among the cutaneous metastases from pancreatic cancer, non-umbilical lesions are more common than umbilical lesions [3]. There are two cases of concurrent umbilical and non-umbilical metastases reported [3].

Umbilical metastases usually arise from the gastrointestinal tract in men and from the ovaries in women [4]. In addition to the “soil and seed” hypothesis [5] and “chemotaxis” postulate [6], several other explanations have been proposed for umbilical metastases. Direct contiguous extension of omental deposits to the anterior peritoneal reflection is possible [7]. The pancreas has copious lymphatic drainage and lymphatic dissemination to the umbilical can occur [8]. Spread may also occur through falciform ligament, medial umbilical ligament of the urachus, vitellointestinal duct remnant, and obliterate vitelline artery [7,9]. Tumor embolization through blood vessels can result in hematogenous spread to the umbilicus [10].

In a case series reported by Yendluri et al. [11], over 90% of pancreatic cancers associated with SMJN arose from the pancreatic body and tail. This could be related to the tendency of tail of pancreas cancers to remain asymptomatic until the advanced stage of the disease, when distant metastasis has already occurred. In contrast, non-umbilical cutaneous metastases are more common in adenocarcinoma involving the head of the pancreas [12] - perhaps due to a predominantly different route of metastatic spread. In general, pancreatic cancers with cutaneous metastases tend to be moderately or poorly differentiated [3], possibly due to these tumor cells expressing a more aggressive, invasive phenotype [13].

To the best of our knowledge, this is the first case of advanced pancreatic cancer presenting as Sister Mary Joseph nodule and concomitant pancreatitis. It is our hypothesis that the primary lesion in the distal pancreas (being relatively asymptomatic) was able to grow large enough to present as cutaneous metastases and cause post-obstructive pancreatitis at the same time. No obvious post-obstructive dilation of the upstream pancreatic duct was seen on endosonography or cross-sectional imaging. Inspissated mucin may have caused the pancreatitis [14]. In a retrospective analysis of patients with pancreatic cancer presenting as acute pancreatitis, six of 47 patients did not have main pancreatic duct dilation [14]. In our patient, either direct extension to the omentum, peritoneum and beyond or lymphatic spread as the primary route for cutaneous metastasis was more likely.

SMJN can be mistaken for peri-umbilical cellulitis or infection. Other diagnostic consideration for umbilical neoplasia includes primary carcinoma, endometriosis, hernia, pyogenic granuloma, mycosis, angioma, teratoma, dermoid cyst, hypertrophic scar. Diagnosis is usually confirmed with a core biopsy or FNA. Immunohistochemical staining may be required to identify the primary cancer. There is at least one case report of a diagnosis of SMJN following a PET-MRI scan in a patient with proven pancreatic adenocarcinoma and rising CA 91-9 levels despite chemotherapy [15].

As seen in our patient, CA 19-9 and CEA tend to be elevated in 70 to 80% of pancreatic cancer patients with cutaneous metastases [3,16].

Therapy for metastatic pancreatic cancer involves combination chemotherapy regimens, including Folfirinox (as used in our patient) or the combination of nab-paclitaxel and gemcitabine. Overall prognosis (in the presence of SMJN) is poor with mean survival of less than a year. Rarely, aggressive surgery and adjuvant therapy can improve survival in patients with SMJN and oligometastatic spread [17]. Tumor seeding during resection is a serious complication and recurrence within the peritoneal cavity can occur following curative surgery [3].

## Conclusions

Though rare, SMJN should be considered in the differential diagnoses of periumbilical skin lesions. Recognition of this lesion requires clinical awareness and high index of suspicion. Prompt identification may avoid delay in diagnosis of an underlying primary cancer and expedite treatment of the primary cancer. SMJN can be the sole presenting clinical sign without any symptoms that pertain to the underlying malignancy. Umbilical and peri-umbilical dermatologic manifestations should be given appropriate consideration and if lesions are unresponsive to standard treatment, skin biopsy can facilitate the diagnosis. Despite the poor prognosis, aggressive surgery including local excision of SMJN and adjuvant chemotherapy can improve survival.
